# Hedgehog/GLI Signaling at the Interface of Sterol Metabolism, Mitochondrial ROS Signaling and Cellular Plasticity

**DOI:** 10.3390/antiox15070905

**Published:** 2026-07-21

**Authors:** Nicolas Jullien, Sabine François

**Affiliations:** Radiobiology Unit, Department of Radiation Effects, French Armed Forces Biomedical Research Institute (IRBA), 91220 Brétigny-sur-Orge, France

**Keywords:** hedgehog signaling, GLI transcription factors, mitochondria, reactive oxygen species, redox homeostasis, NRF2, sterol metabolism, cellular plasticity

## Abstract

Classically, Hedgehog (Hh)/GLI signaling is recognized as a developmental pathway. Increasing evidence indicates that it also contributes to cellular metabolism and adaptation to stress. In this review, we examine the involvement of Hh/GLI signaling in mitochondrial function and redox homeostasis. Mitochondria are major sources of reactive oxygen species (ROS), which act as signaling molecules in cellular adaptation. Hh signaling both influences and responds to ROS production: GLI activity is regulated by redox-dependent mechanisms, and Hh signaling is associated with mitochondrial bioenergetics, dynamics and quality-control pathways. These interactions may contribute to metabolic adaptation in physiological and pathological settings. We also discuss the contribution of sterol metabolism to this regulatory network. Cholesterol and oxysterols modulate Smoothened activation, linking lipid metabolism to mitochondrial function and redox balance. NRF2-dependent antioxidant pathways maintain mitochondrial redox homeostasis, although direct mechanistic crosstalk with Hh/GLI signaling remains incompletely defined. At the tissue level, Hh signaling is involved in responses to irradiation, inflammation, fibrosis, aging and regeneration. Depending on the biological context, pathway activation may support adaptive responses or contribute to tissue dysfunction. Overall, current evidence supports a role for Hh/GLI signaling in mitochondrial redox adaptation through the integration of metabolic and oxidative signals.

## 1. Introduction—Changing the Paradigm

Canonical Hedgehog pathway activation entails relief of Patched-mediated repression of Smoothened (SMO), leading to activation of GLI transcription factors and expression of downstream target genes. Much of the mechanistic framework for this pathway derives from studies of embryonic development and cancer, in which Hh signaling regulates tissue patterning, cell fate and disease progression [1]. Dysregulated Hh activity in adult tissues has also been linked to persistent proliferation, metabolic changes and an immunosuppressive tumor microenvironment [2]. This developmental and oncogenic framework, however, does not capture the full spectrum of Hh functions.

Hh signaling also intersects with cellular metabolism through GLI-dependent regulation of lipid metabolism [3], non-canonical mTORC2 signaling [4] and AMPK-dependent control of GLI1 stability [5]. These mechanisms are examined in detail in Section 2.

Redox biology adds another dimension: controlled ROS act as signaling molecules [6], and mitochondria are central sources of adaptive ROS signals [7].

Evidence indicates a bidirectional relationship between oxidative stress and Hh signaling: oxidative stress may activate non-canonical Hh signaling [8], whereas SHH–JNK–GLI1 signaling can limit ROS generation [9]. The underlying mechanisms are examined in Section 4.

Sterols and mitochondrial lipid metabolism link these processes. Cholesterol and oxysterols directly regulate SMO and control Hh pathway output [10,11]. Mitochondrial cholesterol trafficking involves several complementary pathways. STARD1 is a major mediator of cholesterol transfer from the outer to the inner mitochondrial membrane, particularly in steroidogenic cells, whereas the contribution of TSPO remains debated [12]. Cholesterol enrichment can impair oxidative phosphorylation and respiratory-supercomplex assembly while depleting mitochondrial glutathione and increasing ROS production [13]. Oxysterols therefore provide an additional link between lipid metabolism, immune signaling and redox regulation [14].

Together, these observations position Hh signaling at the interface of sterol metabolism, mitochondrial redox balance and cellular adaptation.

Although substantial progress has been made in developmental biology, metabolism and redox research, the connections among these fields remain incompletely understood. This is particularly true for Hh signaling, whose roles in metabolic regulation and oxidative-stress responses are still being defined. Here, we review evidence linking Hh signaling to mitochondrial metabolism, lipid trafficking and redox homeostasis, and discuss how mitochondrial ROS and oxidative stress influence GLI activity and downstream cellular responses.

## 2. Hedgehog Signaling Beyond Morphogenesis: Integration with Metabolic Checkpoints

The connection between Hh signaling and cellular metabolism is evident at the level of pathway activation. Cholesterol is required for SMO activity and efficient signal transduction [11,15]. In the liver, Hh activity has been linked to lipid metabolism and metabolic gene expression [3,16]. The pathway also intersects with major nutrient-sensing mechanisms. Crosstalk with mTORC1 has been reviewed in several cellular contexts [17], whereas non-canonical Hh signaling can maintain lipid homeostasis through mTORC2 independently of GLI-mediated transcription [4]. Under energetic stress, AMPK restrains Hh pathway activity by promoting GLI1 phosphorylation and destabilization [5]. Metabolic changes accompanying Hh activation have been described in several experimental settings. In cerebellar granule-neuron precursors and SHH-driven medulloblastoma, SHH signaling is associated with increased expression of glycolysis-related proteins and altered glucose utilization [18]. SHH-driven proliferation is also accompanied by NOX4 expression and ROS production that contribute to HIF-1α stabilization under normoxic conditions [19]. More recently, SHH signaling has been linked to iron metabolism in pulmonary fibroblasts: iron and ROS suppress FOXF1, whereas SHH increases intracellular iron, fibroblast proliferation and SHH secretion [20].

**Figure 1:**
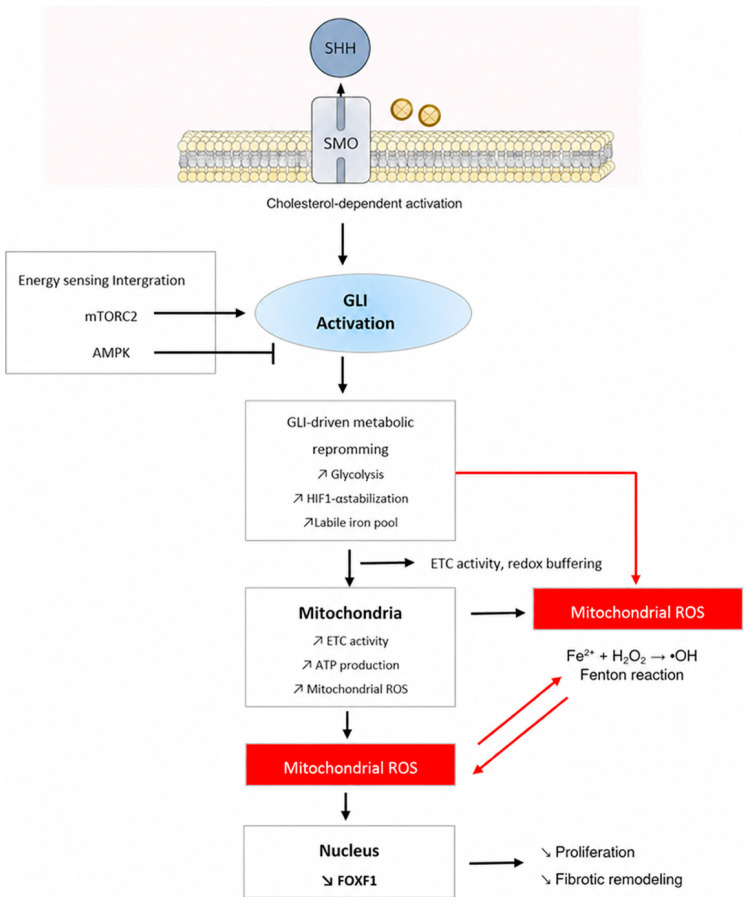
Proposed relationships among Hedgehog signaling, iron metabolism, mitochondrial function and redox homeostasis. Sonic Hedgehog (SHH)-dependent and cholesterol-sensitive activation of Smoothened (SMO) promotes GLI-dependent transcription. mTORC2 is shown as a GLI-independent, non-canonical effector of Hh signaling, whereas AMPK negatively regulates GLI1 stability. Reported downstream effects of Hedgehog pathway activation include increased glycolysis, HIF-1α stabilization and expansion of the labile iron pool. The mitochondrial box summarizes reported changes in electron transport chain (ETC) activity, ATP production and mitochondrial reactive oxygen species (ROS) generation. Iron-dependent ROS produced through Fenton chemistry may further contribute to FOXF1 repression and to proliferative and profibrotic responses. The scheme is presented as a working model, as several of these interactions remain context-dependent and mechanistically unresolved. Black arrows indicate canonical signaling or associated metabolic consequences, whereas red arrows indicate ROS-related interactions.

These observations suggest reciprocal interactions between Hh signaling and cellular metabolism, but their biological significance and causal hierarchy remain incompletely defined. Hh-associated changes in cholesterol metabolism, nutrient sensing, iron homeostasis and ROS production may be functionally interconnected, although the extent to which they drive downstream responses or feed back on pathway activity is likely to depend on the cellular and pathological context. The emerging link between iron-dependent ROS production, FOXF1 repression and tissue remodeling supports a possible SHH–iron–ROS axis. This axis should, however, be regarded as a working model rather than an established regulatory pathway. As illustrated in Figure 1, it provides a conceptual framework for investigating how metabolic and oxidative conditions shape Hh-dependent responses.

## 3. Mitochondria as Signaling Platforms: ROS as Second Messengers

Although traditionally viewed as the primary source of cellular ATP, mitochondria also sense and transmit metabolic and environmental information. A major component of this signaling activity is the generation of ROS [7]. Mitochondrial ROS, produced mainly at complexes I and III of the electron transport chain, function as second messengers in oxygen sensing and other adaptive responses [21,22,23]. The mitochondrial redox state can alter cysteine oxidation in redox-sensitive proteins, thereby changing protein activity, localization and molecular interactions [24]. HIF-1α stabilization during hypoxia is a well-characterized example: complex III-derived ROS contribute to prolyl-hydroxylase inhibition and activation of hypoxia-responsive transcription. These responses nevertheless vary with cell type, metabolic state and stimulus intensity.

Mitochondrial ROS signaling does not occur in isolation. NADPH oxidases (NOX) generate superoxide or hydrogen peroxide in a regulated manner and contribute to signal transduction [25,26]. Crosstalk between NOX-derived ROS and mitochondrial ROS can amplify redox signals through processes commonly described as ROS-induced ROS release [27,28].

**Figure 2:**
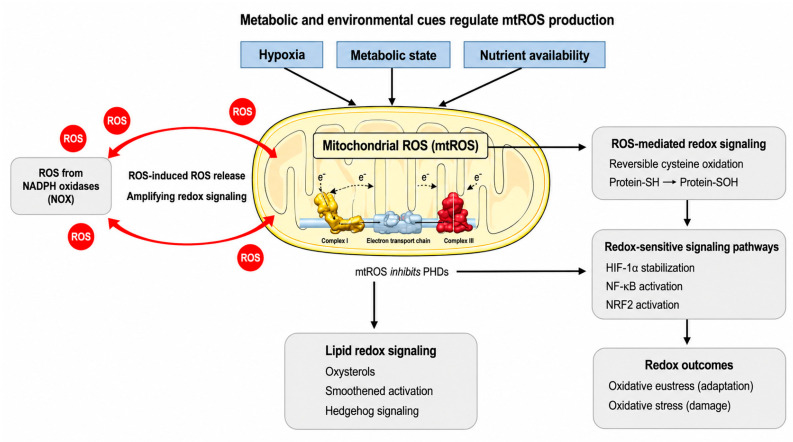
Mitochondria as central hubs of redox signaling. Mitochondrial ROS (mtROS) are produced mainly at complexes I and III of the electron transport chain and are modulated by oxygen availability, nutritional status and cellular metabolic activity. Changes in mtROS production alter the redox status of cysteine residues in target proteins and may affect many signaling cascades. Hypoxia inhibits prolyl hydroxylases, which stabilize HIF-1α. Likewise, NF-κB and NRF2 signaling are responsive to changes in cellular redox state. Crosstalk with NADPH oxidases (NOX) may additionally enhance ROS production. Simultaneously, oxidation of lipids produces oxysterols that can activate Hedgehog signaling via Smoothened. The biological ramifications of these signaling events depend on the cellular environment and the extent of the redox challenge.

Mitochondria can also influence cell behavior through oxidized lipids. Oxysterols are particularly relevant because several members of this class bind or modulate SMO and regulate Hh-dependent transcription [11]. Alterations in lipid oxidation or sterol availability may therefore affect processes ranging from development to tissue repair. Conversely, Hh signaling is sensitive to cellular metabolic and redox state, supporting a bidirectional relationship between mitochondrial activity and adaptive signaling. These mitochondrial redox and lipid-mediated signaling interactions are summarized in Figure 2.

## 4. Redox Regulation of GLI Activity

In adult tissues, GLI activity remains responsive to environmental cues, including hypoxia and oxidative stress. Hypoxia-associated activation of Hh/GLI signaling has been described in several cancer models [8,29]. In human brain organoids, Hh pathway activation has also been reported to protect against hypoxia-induced ferroptotic injury [30].

Beyond hypoxia, the cellular redox state can influence GLI expression, stability and transcriptional activity. In myeloid cells, the FOXO1–β-catenin axis modulates Hh/GLI1 signaling during oxidative stress [31]. ROS should not, however, be regarded only as inhibitory signals. In SHH-driven cerebellar progenitors, NOX4-derived ROS contribute to HIF-1α stabilization and proliferative signaling [19]. The effect of ROS on Hh/GLI signaling therefore depends on their abundance, subcellular source and cellular context.

When oxidative stress becomes excessive, Hh/GLI signaling may become destabilized. In stem Leydig cells, increased matrix stiffness induces Piezo1-dependent Ca^2+^ influx, mitochondrial dysfunction and excessive ROS production, leading to GLI1 degradation through the ubiquitin–proteasome system [32]. Ferroptotic stress is characterized by iron-dependent lipid peroxidation and impaired antioxidant defenses. In colorectal cancer, berberine-induced inhibition of the Gli1/STAT3 axis reduced GPX4, SLC7A11 and FTH1 expression and increased ferroptotic susceptibility [33]. In hepatocellular carcinoma cells, hypoxia-associated oxidative stress has instead been linked to non-canonical Hh activation and a more aggressive phenotype [8]. The cited hepatocellular carcinoma study did not demonstrate an association between non-canonical Hh activation and reduced GLI activity. More broadly, GLI transcription factors may also be activated independently of SMO [34].

The molecular basis of this redox-dependent transition remains incompletely defined. No specific redox-sensitive cysteine residue in GLI1 has yet been functionally established as a direct molecular switch controlling its activity. Current evidence instead points to indirect regulation through redox-sensitive signaling and protein turnover. GLI1 stability is also regulated by the E3 ubiquitin ligases β-TrCP and ITCH in developmental and tumor contexts [35,36]. However, whether either ligase mediates GLI1 degradation specifically in response to excessive oxidative stress, and which upstream ROS-responsive kinase or GLI1 degron is involved, remains unresolved.

Taken together, these studies indicate that ROS do not regulate Hh/GLI signaling through a simple binary switch. Rather, pathway output is shaped by the intensity, duration, subcellular origin and cellular context of ROS production. Moderate and spatially controlled redox signaling may sustain Hh/GLI activity, whereas prolonged or excessive oxidative stress can promote GLI destabilization and disrupt downstream transcriptional responses. This non-linear regulation may be particularly relevant in chronic pathological settings, where persistent redox imbalance can influence tissue remodeling, regenerative capacity and cell fate.

## 5. Hedgehog Signaling in Mitochondrial Homeostasis and Stress Responses

Evidence from several experimental systems links Hh signaling to mitochondrial bioenergetics, dynamics and quality-control pathways.

One of the clearest demonstrations of a relationship between Hh signaling and mitochondrial function comes from neuronal models. SHH pathway activation increased mitochondrial abundance, oxidative phosphorylation and respiratory activity in hippocampal neurons, and was associated with altered mitochondrial morphology and greater resistance to mitochondrial stress [37]. These findings indicate that Hh activation can influence mitochondrial fitness, although the extent to which this reflects direct mitochondrial regulation or broader metabolic adaptation remains uncertain.

Related effects have been reported in other systems. In macrophages, SHH stimulation promotes oxidative phosphorylation and efferocytosis during scar formation [38]. In the Drosophila wing disk, by contrast, Hh signaling enhances glycolytic ATP production [39]. These observations argue that Hh signaling is associated with metabolic flexibility rather than with a single bioenergetic state.

Mitochondrial quality-control mechanisms are essential during stress, and several studies link Hh signaling to autophagy, mitophagy and mitochondrial dynamics. In hepatocellular carcinoma cells, inhibition of Hh signaling induces BNIP3-dependent autophagy [40]. In clear-cell renal-cell carcinoma, GLI2-dependent Parkin expression promotes mitophagy and pazopanib resistance [41]. Hh signaling has also been linked to DRP1-dependent mitochondrial dynamics in a cardiac-hypertrophy model [42]. Because these findings arise from distinct disease models, they support an association with mitochondrial quality control but do not yet define a conserved mechanism.

A common feature of these studies is the close association between mitochondrial function, oxidative stress and inflammatory responses. In the mammary tumor microenvironment, Hh signaling regulates the metabolism and polarization of tumor-associated macrophages [43]. Hh-dependent autophagic responses can also support survival and chemoresistance under adverse conditions [44]. These data are consistent with functional interactions among Hh signaling, mitochondrial homeostasis and stress-response pathways, but the causal hierarchy remains model-dependent.

These mechanisms are relevant to radiation exposure, where persistent mitochondrial dysfunction [45] and mitochondrial ROS production [46] shape tissue responses. Their implications are examined in Section 6.1.

Taken together, these studies link changes in Hh pathway activity to alterations in mitochondrial metabolism, turnover and stress responses. However, these effects are highly context-dependent, and it remains unclear whether they reflect direct regulation by Hh signaling or secondary adaptive responses. This question is particularly relevant in conditions of sustained oxidative stress, including tissue injury and irradiation, in which mitochondrial dysfunction and persistent ROS production contribute to long-term tissue remodeling.

## 6. Implications for Tissue Stress Adaptation

At the tissue level, the interactions described above vary according to the tissue, the type of injury and the duration of pathway activation. The following subsections examine their implications for irradiation, inflammation, fibrosis, aging and regeneration.

### 6.1. Irradiation and Radiosensitivity

Ionizing radiation alters mitochondrial function, causing impaired oxidative phosphorylation, increased ROS production and persistent oxidative stress [45,47]. In irradiated salivary glands, SHH gene delivery reduced cellular senescence, promoted DNA repair and decreased oxidative stress [48]. Mitochondrial ROS may also sustain feedback loops that amplify injury and influence radiosensitivity [46]. These observations suggest that Hh-dependent responses may modify radiation outcome through effects on repair and redox homeostasis, but they do not yet establish a universal mechanism across tissues.

### 6.2. Inflammation and Immunometabolic Regulation

Mitochondrial metabolism is closely coupled to immune-cell activation and inflammatory signaling. As discussed in Section 5, Hh activity alters tumor-associated macrophage metabolism and polarization [43]. In pulmonary fibrosis, epithelial SHH promotes OPN-dependent alternative macrophage activation [49]. Together, these findings illustrate how Hh signaling can shape immunometabolic responses through distinct tissue-specific mechanisms.

Mechanistically, myeloid GLI1 has been linked to suppression of NEK7/NLRP3 inflammasome activation during oxidative stress, thereby limiting IL-1β maturation [31]. This example illustrates how Hh/GLI signaling can connect redox sensing to inflammatory output, although the direction and magnitude of the effect remain tissue and stimulus dependent.

### 6.3. Fibrosis and Tissue Remodeling

Hh signaling is activated in several fibrotic disorders and contributes to tissue remodeling beyond its physiological role in repair. Sustained pathway activity is associated with fibroblast activation, extracellular-matrix deposition and progressive fibrosis [50]. In systemic sclerosis fibroblasts, GLI2/Hh and cJUN/AP-1 signaling mutually reinforce one another [51]. The macrophage-mediated pulmonary mechanism is discussed in Section 6.2 [49], whereas in the liver, GLI2 contributes to hepatic stellate-cell activation through TGF-β-related signaling [52]. Together, these studies support organ-specific mechanisms of chronic Hh/GLI-dependent remodeling.

### 6.4. Aging and Mitochondrial Decline

Mitochondrial dysfunction is a hallmark of aging and contributes to the progressive loss of tissue homeostasis [53]. Direct evidence that Hh/GLI signaling drives age-related mitochondrial decline remains limited. The matrix-stiffness-dependent loss of GLI1 described in Section 4 provides one age-related example [32]. In skeletal muscle, GLI3 and DHH regulate stem- and progenitor-cell programs relevant to regeneration, but these studies do not establish a direct mitochondrial mechanism [54,55].

### 6.5. Muscle Regeneration and Tissue Repair

Successful skeletal-muscle regeneration requires mitochondrial remodeling and metabolic adaptability. Mitochondrial fission, fusion and mitophagy help preserve muscle stem-cell regenerative competence [56,57]. AMPK-dependent glycolytic reprogramming also contributes to satellite-cell activation [58]. Hh signaling regulates muscle stem-cell activation and self-renewal through GLI-dependent mechanisms [54] and influences fibro-adipogenic progenitor fate [55,59]. After high-dose irradiation, persistent oxidative stress and mitochondrial dysfunction may constrain regeneration, and pharmacological modulation of Hh signaling has been explored as a means of improving muscle repair [60,61].

### 6.6. Integrative View of Hh-Dependent Stress Adaptation

**Figure 3:**
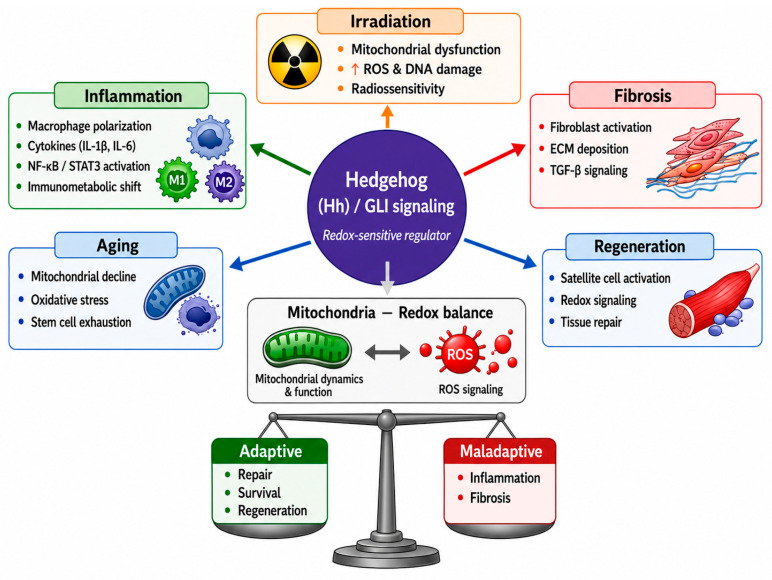
Proposed relationship between Hh/GLI signaling, mitochondrial function and redox homeostasis during tissue adaptation. The scheme integrates the contexts discussed in Section 6—irradiation, inflammation, fibrosis, aging and regeneration—and illustrates how mitochondrial dynamics and reactive oxygen species (ROS) may contribute to adaptive tissue repair or to maladaptive outcomes, including chronic inflammation, fibrosis and tissue dysfunction.

Alterations in Hh/GLI signaling have been associated with changes in mitochondrial function and redox homeostasis in diverse settings, including irradiation, inflammation, fibrosis, aging and tissue regeneration. Current evidence suggests that these interactions contribute to cellular and tissue responses to metabolic and oxidative stress, although their molecular basis remains incompletely understood. Their biological consequences are highly context-dependent: they may support tissue repair and regeneration under controlled conditions, but contribute to chronic inflammation, fibrosis and tissue dysfunction when signaling or oxidative stress persists. Figure 3 summarizes these proposed relationships and their potential adaptive or maladaptive outcomes.

## 7. Future Perspectives and Therapeutic Implications

Mounting evidence links Hh/GLI signaling to mitochondrial function and redox control, but several fundamental questions remain. It is still unclear whether the mitochondrial changes observed after Hh activation are direct consequences of GLI-dependent transcription or indirect consequences of broader metabolic and stress-response programs. The hierarchy among Hh/GLI, ROS, TGF-β, Notch and antioxidant pathways also remains poorly defined. NRF2 is central to mitochondrial redox buffering, yet direct mechanistic crosstalk between NRF2 and Hh/GLI has not been established consistently across models. Future work should therefore prioritize causal experiments that distinguish pathway-specific effects from secondary adaptation and define the relevant cellular and subcellular sources of ROS.

At the tissue level, Hh signaling influences communication among immune cells, stromal populations and stem or progenitor cells. These interactions may be important in tissue repair, chronic inflammation and fibrosis, where mitochondrial dysfunction and oxidative stress shape disease progression. From a translational perspective, Hh pathway modulation is likely to remain highly context-dependent. SMO inhibition is clinically effective in tumors driven by constitutive Hh activation, such as advanced basal-cell carcinoma (NCT00957229), whereas trials in pancreatic and colorectal cancer have not shown comparable benefit (NCT01088815; NCT00636610). Emerging cell-free approaches may also modulate Hh signaling indirectly. MSC-derived extracellular vesicles have been reported to attenuate liver and renal fibrosis through effects on Hh/SMO signaling [62,63], although their relationship with mitochondrial redox restoration remains unknown. Whether these effects also involve direct restoration of mitochondrial function remains to be established.

Resolving these mechanisms across tissues and disease states will help identify the biological contexts in which Hh pathway modulation may be therapeutically beneficial.

## Data Availability

No new data were created in this study. The review is based on previously published literature, all of which is cited in the manuscript and publicly available through scientific databases.
